# Supplementary material for: Primer synthesis by a eukaryotic-like archaeal primase is independent of its Fe-S cluster
Source: Nat Commun. 2017 Nov 23;8:1718. doi: 10.1038/s41467-017-01707-w (PMC5700102; doi:10.1038/s41467-017-01707-w)
Supplement: Supplementary file 1 — Supplementary Information [file 41467_2017_1707_MOESM1_ESM.pdf]

a

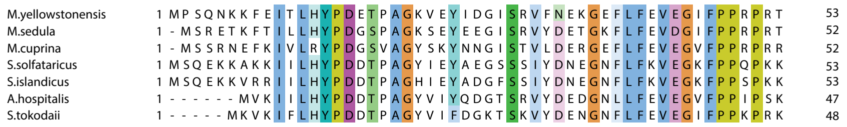

b

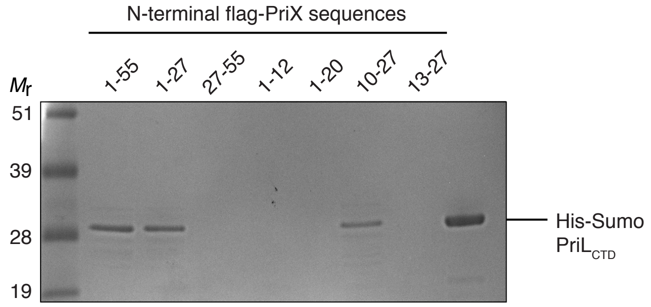

Supplementary figure 1. The N-terminal region of PriX interacts with the PriL-CTD. (**a**) Multiple sequence alignment of PriX N-termini of different archaeal PriX sequences. In the alignment, conserved amino acids are coloured according to chemical character. (**b**) Pull-down experiments of His-Sumo-PriL-CTD with various flag-tag constructs of the PriX N-terminus, immobilised on beads.

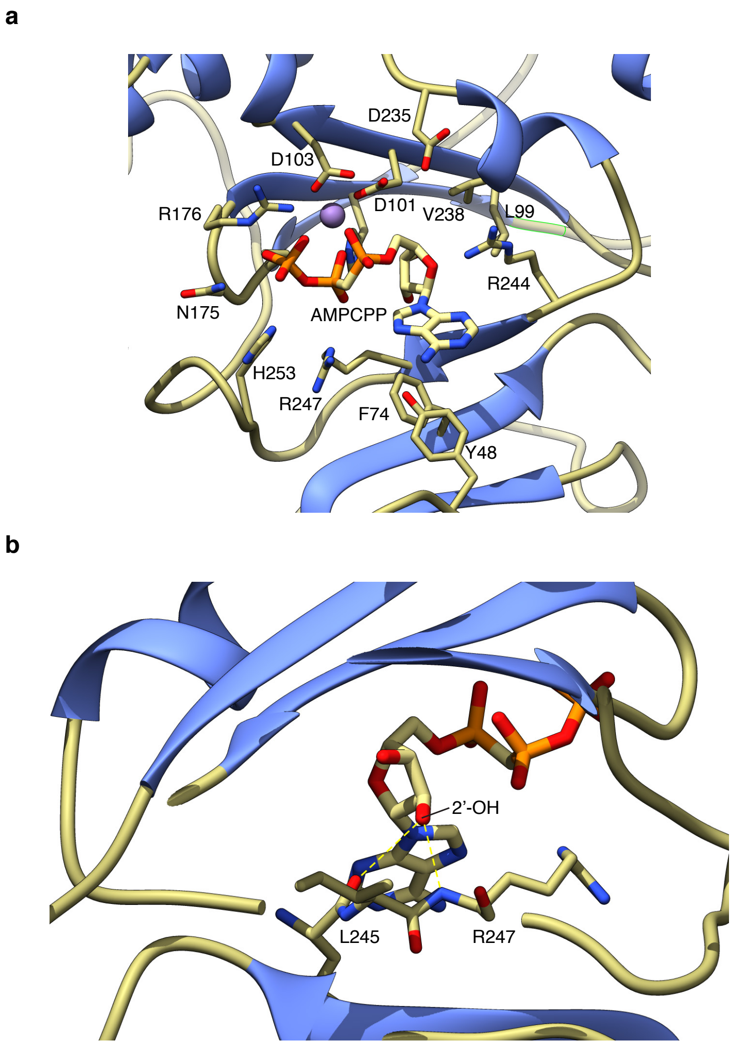

Supplementary figure 2. The PriS active site with bound non-hydrolysable nucleotide analogue AMPCPP. (**a**) View of the PriS active site, showing the amino acids involved in binding AMPCPP. (**b**) The 2'-hydroxyl of the AMPCPP ribose makes two hydrogen bonds to the main-chain atoms of L245 and R247.

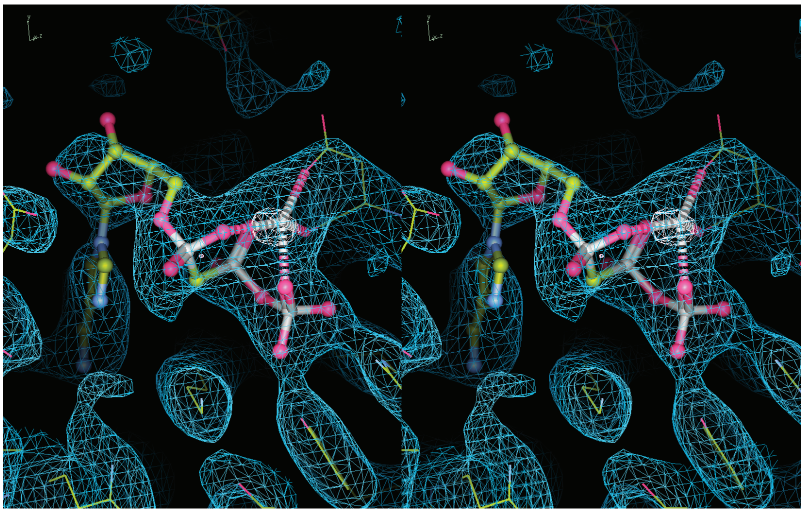

Supplementary figure 3. Stereo view of the sharpened $2F_o$-F_c electron density map for the PriX-bound AMPCPP, contoured at 1 rmsd and coloured in blue. The anomalous difference map for the Mn^{2+} ion, contoured at 6 rmsd, is also shown in pink. The figure was produced in Coot (Emsley & Cowtan, 2004).

a

```
Sulfolobus_acidocaldarius          1 -----MEENRKRKRIILHYPDDTPAGYIEFDGRVSKIYNEKGEFIFQVEGSFPPIPRK-  53
Metallosphaera_yellowstonensis     1 MRGREVPSQNKKFEITLHYPDETPAGKVEYIDGISRVFNEKGEFLFEVEGIFPPRPRT-  58
Sulfolobus_solfataricus            1 -----MSQEKKAKKIILHYPDDTPAGYIEYAEGSSSIYDNEGNFLFKVEGKFPPQPKKS  54
Acidianus_hospitalis               1 -----------MVKIILHYPDDTPAGYVIYQDGTSRVYDEDGNLLFEVEGVFPPIPSK-  47
Aeropyrum_camini                   1 ------MGVDEAYQALLRACGER--GFEECRDAYRMFLEEACREAGSCPKRRSPGTGQ-  50
Acidilobus_saccharovorans          1 -----------MFAEAAQRCEESP---AECPQIAESILRTMCPNVNMCS--VTAVKSR-  42
Caldisphaera_lagunensis            1 ----------MVFEDVINNCQRSE---IDCQKSIKELLKKYCPNPSDCSISIYRENLV-  45
Aeropyrum_pernix                   1 ------MGVNEAYEALLRACGDG--DFEECRSGYQRFLEEACREAGTCPKRRSSGAGR-  50

Sulfolobus_acidocaldarius         54 VNYDWIDKILEKGLPDARKRFILYVASRYLVNVKGLSEEDVVKILVDFYGRH-----GG 107
Metallosphaera_yellowstonensis    59 SSMEWIDKVLEKGLKDGRKRFILYVASRYLLNVKGLTEEETVERLKEFYYK------GG 111
Sulfolobus_solfataricus           55 SDYSWIEKVLEMGLQDSRKRFILYVASRYLVNVKGVNEDEALQTLKEFYYKL-----QS 108
Acidianus_hospitalis              48 VNYSWIDTILEKGLPDGRKRFILYVASRYLVNVKNLNEDEALEKLKEFYYKT-----GS 101
Aeropyrum_camini                  51 GRYAWVEAVLRSGVPDGRSRLILYVISRYLVNVKGLEPGEAEAVIEEFLRVCCERHGNC 109
Acidilobus_saccharovorans         43 GDFSWIESVLSTGVPDGRHRLILYVLSRYLANVKGLNEADAVNEIRGFLERSCKNFGNC 101
Caldisphaera_lagunensis           46 QKFNWIEKIIKNGVPDGRTRLILYVISRYLINVKKLNDEEALNQINEFINNSCKNYNNC 104
Aeropyrum_pernix                  51 GKYVWVESIIRSGVPDGRSRLILYVISRYLVNVKGLEPGEAEAVIDEFLRVCCEKHGNC 109

Sulfolobus_acidocaldarius        108 SKVYESWIRSVVRGVKSKNLKPWSLKKIESNDKEMYELILKVLNS-------------- 152
Metallosphaera_yellowstonensis   112 GRVYDTWLRSVVRGVKTKGLRPPSLRTLELKDKELYLEIKKVLEENS------------ 158
Sulfolobus_solfataricus          109 GKVYESWLKSVINGVKKKGLLPWSLKRIEERDKEMYNEIIRVLKNS------------- 154
Acidianus_hospitalis             102 GKIYDAWIRSVIKGVKTKGLRPPSLKKLQEKDPELYEEIRRVLG--------------- 145
Aeropyrum_camini                 110 RKIYKSWIRNVLRRVKEGGWRPWTLERIKSEDPELYSIIESIISGGGG----------- 157
Acidilobus_saccharovorans        102 SKVYDSWIRNVVSKVKSGGWKPWSLEKLKEKDPDLYNTVLKLISESGKGQVDTLSATRS 160
Caldisphaera_lagunensis          105 SQIYKSWIINVIGKVKNGKWKPWNMDKIRNSDPELYKVLEPIIGKNN------------ 151
Aeropyrum_pernix                 110 RKIYKSWIRNVLRRVREGGWRPWTLERIRSEDPELYRIIEPIVSAGGG----------- 157
```

b

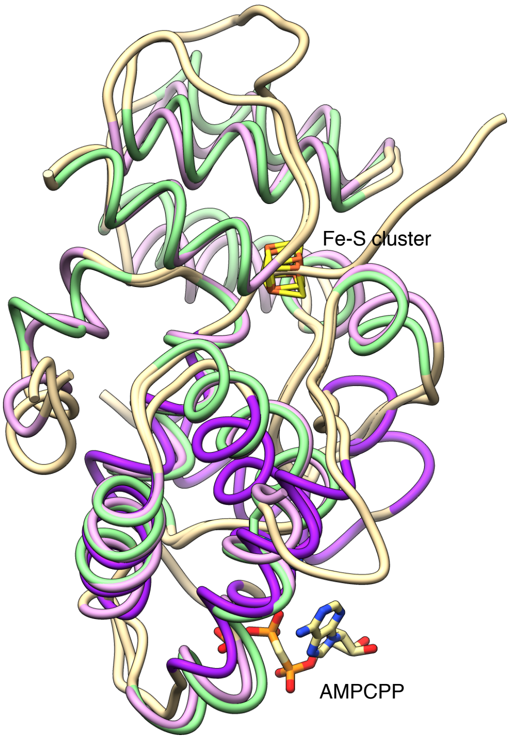

Supplementary figure 4. (**a**) Multiple-sequence alignment of archaeal PriX sequences. The position of NTP-binding amino acids D70, R72 and R74 of *S. solfataricus* primase are highlighted by red boxes. (**b**) Superposition of the AMPCPP-bound PriX structure (purple) with the yeast (green; PDB ID 3LGB) and human (pink; PDB ID 3Q36) PriL-CTD structures.

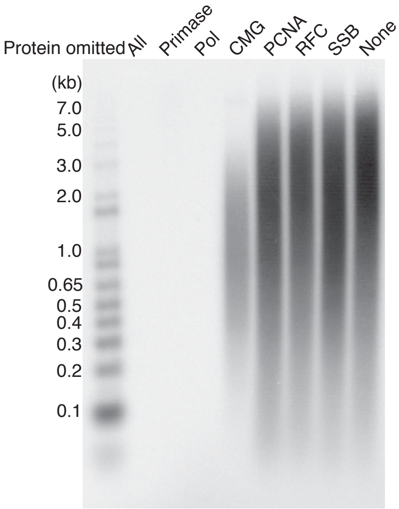

Supplementary figure 5. Reconstitution of a primase-dependent M13-based replication assay. The full reconstitution reaction (right-hand lane) contained M13mp18 ssDNA (5 ng; New England Biolabs), 24 nM MCM, 140 nM Cdc45-GINS, 100 nM PCNA, 50 nM RFC, 50 nM PolB1-HE, 400 nM SSB and 50 nM primase. The proteins listed above individual lanes were omitted from the reactions that were subsequently electrophoresed in those lanes.

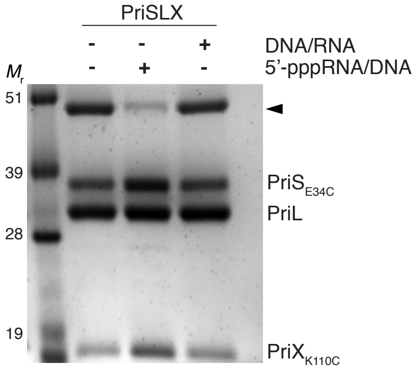

Supplementary figure 6. BMOE crosslinking of PriSLX double-mutant $PriS_{E34C}$, $PriX_{K110C}$, in the presence of RNA/DNA and 5'-pppRNA/DNA. The products of the crosslinking reactions were separated by SDS-PAGE and stained with Coomassie Blue. The arrowheads mark the position of the crosslinked product in the gel.

a

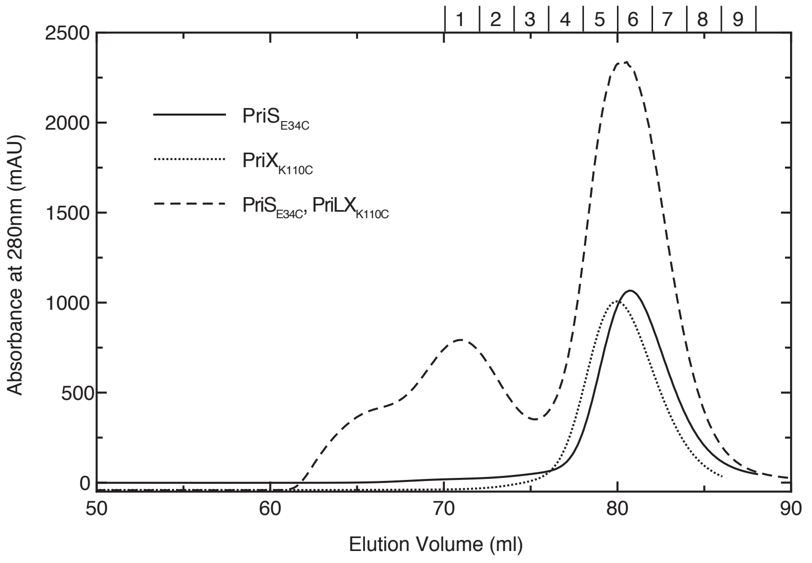

b

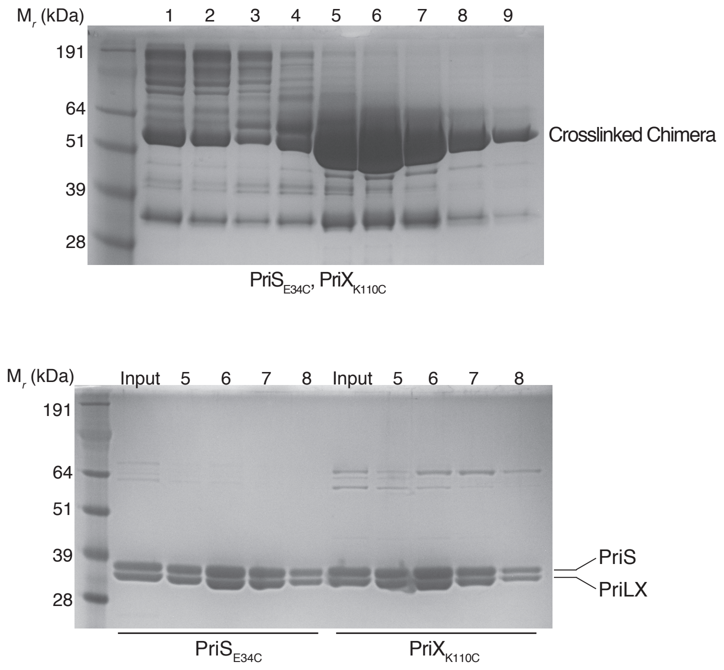

Supplementary figure 7. Preparation of crosslinked Chimera (PriS$_{E34C}$ - PriLX$_{K110C}$). (**a**) Gel-filtration chromatography of BMOE-crosslinked PriS$_{E34C}$ - PriLX$_{K110C}$ and single-mutants PriS$_{E34C}$ – PriLX, PriS - PriLX$_{K110C}$ (controls). (**b**) SDS-PAGE analysis of relevant gel-filtration fractions for crosslinked PriS$_{E34C}$ - PriLX$_{K110C}$ (top) and single-mutants PriS$_{E34C}$ - PriLX, PriS - PriLX$_{K110C}$ (bottom).

a

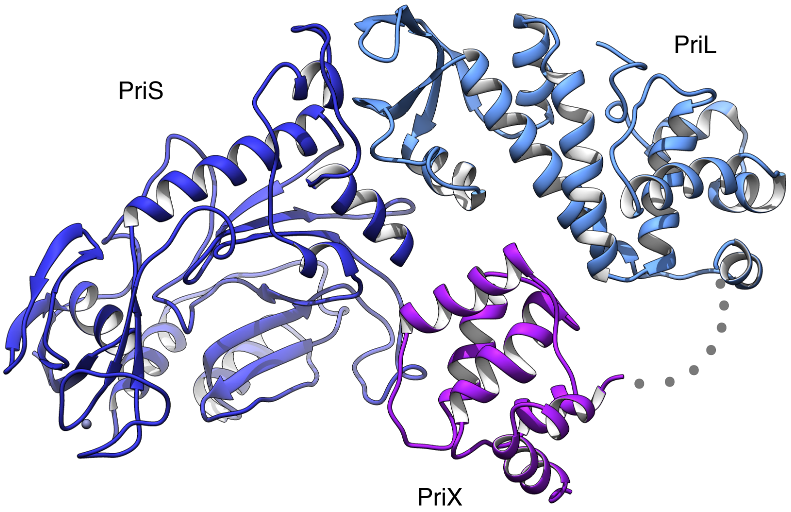

b

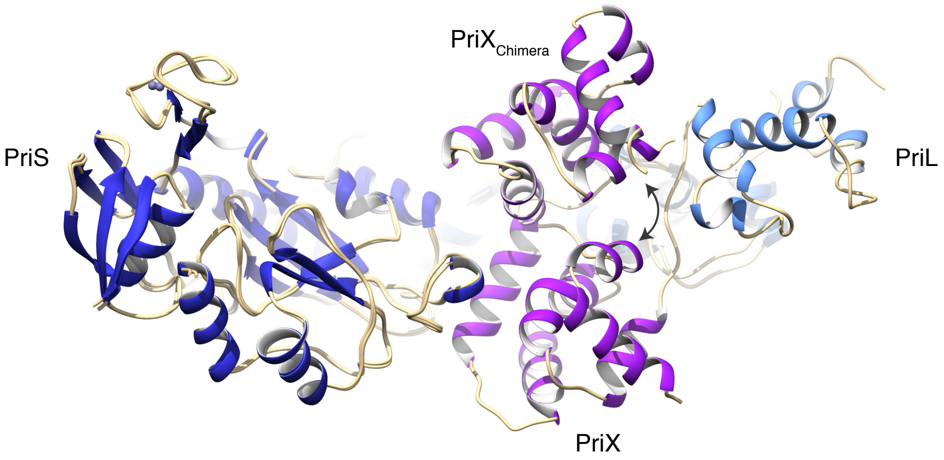

Supplementary figure 8. Crystal structure of Chimera. (**a**) Side view of the Chimera structure, colour-coded as PriSLX in Figure 1. The disordered linker region between PriL and PriX is drawn as gray dots. (**b**) Superposition of PriSLX and Chimera, highlighting the different position taken up by PriX in the two structures.

Emsley, P., & Cowtan, K. (2004). Coot: Model-building tools for molecular graphics. *Acta Crystallogr D Biol Crystallogr*, *60*, 2126–2132.
